# Gut Microbiota Imbalance in Metastatic Colorectal Patients Treated With EGFRI and Long-Term Antibiotic Therapy for Cutaneous Toxicity: A Pilot Study

**DOI:** 10.7759/cureus.25007

**Published:** 2022-05-15

**Authors:** Cristina-Maria Popa, Simona-Laura Ianosi, Stefania-Cornelia Dorobantu, Adrian Saftoiu

**Affiliations:** 1 Department of Dermatology, Emergency County Hospital, Craiova, ROU; 2 Genetics, Regional Center for Medical Genetics, Craiova, ROU; 3 Gastroenterology and Hepatology, Research Center of Gastroenterology and Hepatology, Craiova, ROU

**Keywords:** gut dysbiosis, egfri, colorectal cancer, gut microbiome, skin toxicity

## Abstract

Patients with metastatic colorectal cancer (mCRC) frequently experience epidermal growth factor inhibitors (EGFRI)-induced skin side effects. Antibiotic treatment with doxycycline is often required in order to manage the skin and mucosal toxicity. Since these patients already have significant gut dysbiosis, the long-term antibiotic treatment may destabilize their gut microbiome.

Objectives

The assessment of intestinal dysbiosis in patients undergoing treatment with EGFRI, who require antibiotic treatment with doxycycline in order to manage adverse skin effects.

Methods

We conducted a prospective pilot study between 2020 and 2021 involving 10 patients with mCRC. These patients were undergoing treatment with EGFRI and required either short-term or long-term treatment with doxycycline in order to manage skin toxicity.

Results

The patients with mCRC who were treated with doxycycline for 8 weeks showed overexpression of Escherichia coli, Candida, and Geotrichum species compared to the patients who only received doxycycline treatment for two weeks.

Conclusions

The elevated levels of Escherichia coli and Candida species in the patients who received doxycycline for eight weeks compared to the patients who received the treatment for two weeks could provide a starting point for the development of a standardized guideline regarding the use of pre-active or reactive antibiotic treatment. We also highlight the importance of analyzing the intestinal microbiome of these patients. The identification of overexpressed species, as well as the deficiency of certain protective species, could guide the administration of probiotics to cover and repair the affected intestinal flora.

## Introduction

The emergence of molecularly targeted therapies, in particular epidermal growth factor inhibitors (EGFRI), has been an important upgrade in oncological treatments. Although they are characterized by unique adverse effects, being dominated by the occurrence of cutaneous toxicity, particularly the papulopustular rash, they have much lower systemic adverse effects compared to classical chemotherapy [1].

Tetracycline treatment is usually chosen to treat post-EGFRI papulopustular rashes [2-3]. The lack of antibiotic treatment can worsen the skin reactions, progressing to severe complications or superinfections, which could require the patients to reduce the dose or even discontinue the cancer treatment [4-5].

However, since colorectal cancer (CRC) patients already have significant gut dysbiosis, the use of long-term antibiotic treatments may further destabilize their gut flora [2].

The human microbiome is in a permanent symbiotic relationship with the host, playing an important role in the maintenance of the metabolic homeostasis, human physiology, digestion, and detoxification processes, as well as in the development of the immune system [6].

Intestinal dysbiosis occurs as a consequence of the qualitative and quantitative changes in the gut microbiota [7-8]. The close connection between intestinal dysbiosis and colorectal cancer highlights the importance of assessing the microenvironmental markers when screening for early colorectal cancer [9].

The disturbances of the microbiota may be promoters in the development of colorectal cancer; however, the ecosystem imbalances may also be a consequence of the anticancer therapy [10].

Given the important role played by the Fusobacterium nucleatum in the physiology and pathology of colorectal cancer, the use of antibiotics whose spectrum targets this bacterial species may benefit CRC patients significantly. Although there are no official guidelines for treating Fusobacterium nucleatum infections, according to the Sanford 2020 guidelines, the oral use of tetracycline-type bacteriostatic agents is extremely useful in the treatment of chronic Fusobacterium spp infection. Therefore, the use of doxycycline in patients under EGFRI treatment has a dual role: management of therapy-induced cutaneous rash and action against Fusobacterium [2,11].

The aim of this study is to assess the impact of the doxycycline-based antibiotic treatment on the microbiome of colorectal cancer patients undergoing EGFRI therapy. We also analyzed and compared, for the first time, the gut dysbiosis between two groups of colorectal cancer patients, who received short- or long-term doxycycline treatment, respectively.

## Materials and methods

Case selection

The study was conducted in agreement with the local ethics committee and in accordance with the Helsinki Declaration. This study included 10 colorectal cancer patients from St. Nectarie Medical Centre undergoing EGFRI treatment (Cetuximab and panitumumab), as well as doxycycline-based treatment, for the management of EGFRI-induced skin toxicity between 2021-2022.

The 10 patients were divided into two study groups; patients from the first group received doxycycline treatment for less than two weeks while patients from the second group received doxycycline treatment for longer periods (between six and eight weeks).

**Figure 1:**
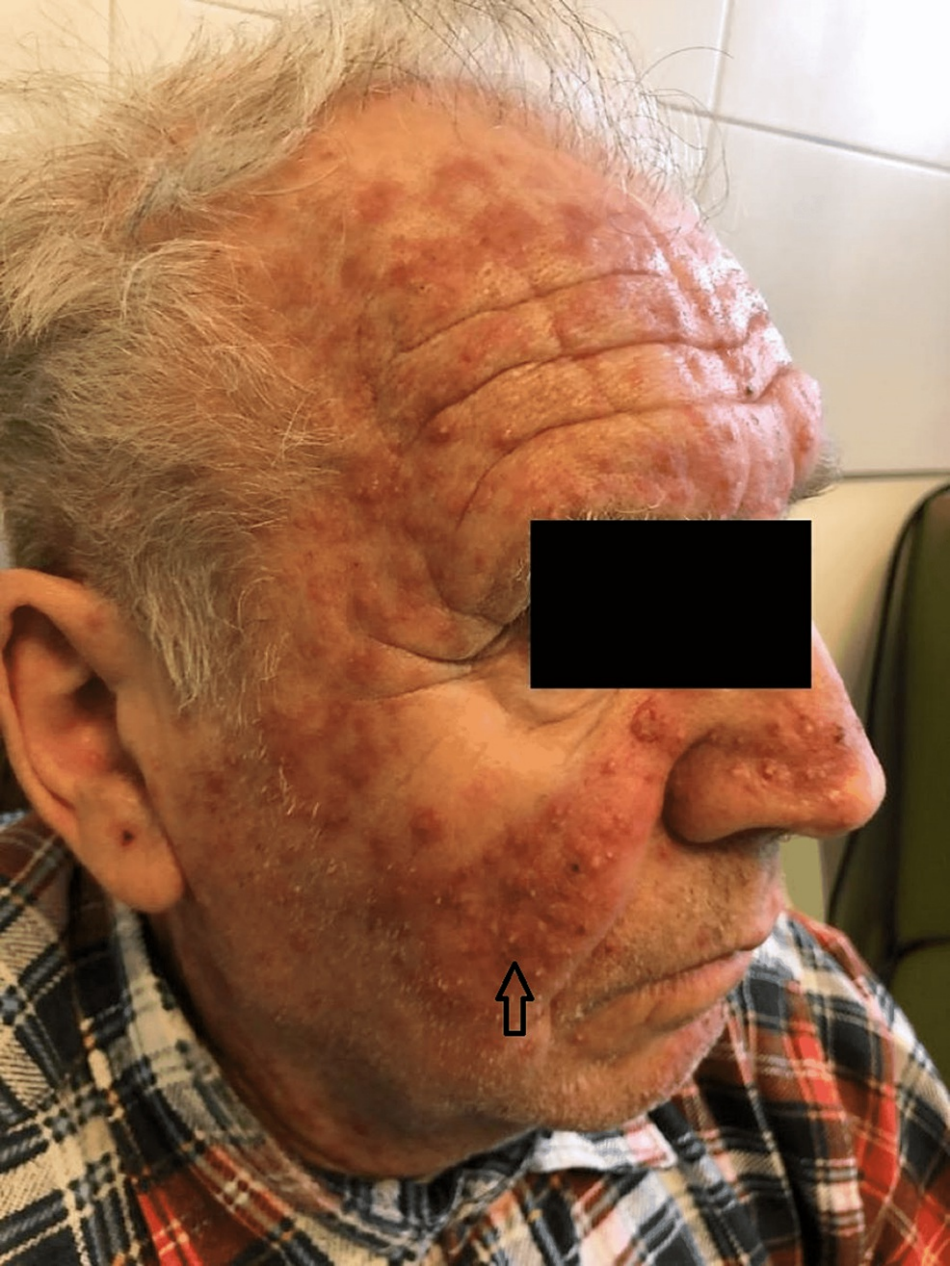
Papulopustular rash, erythema, hematic crusts

**Figure 2:**
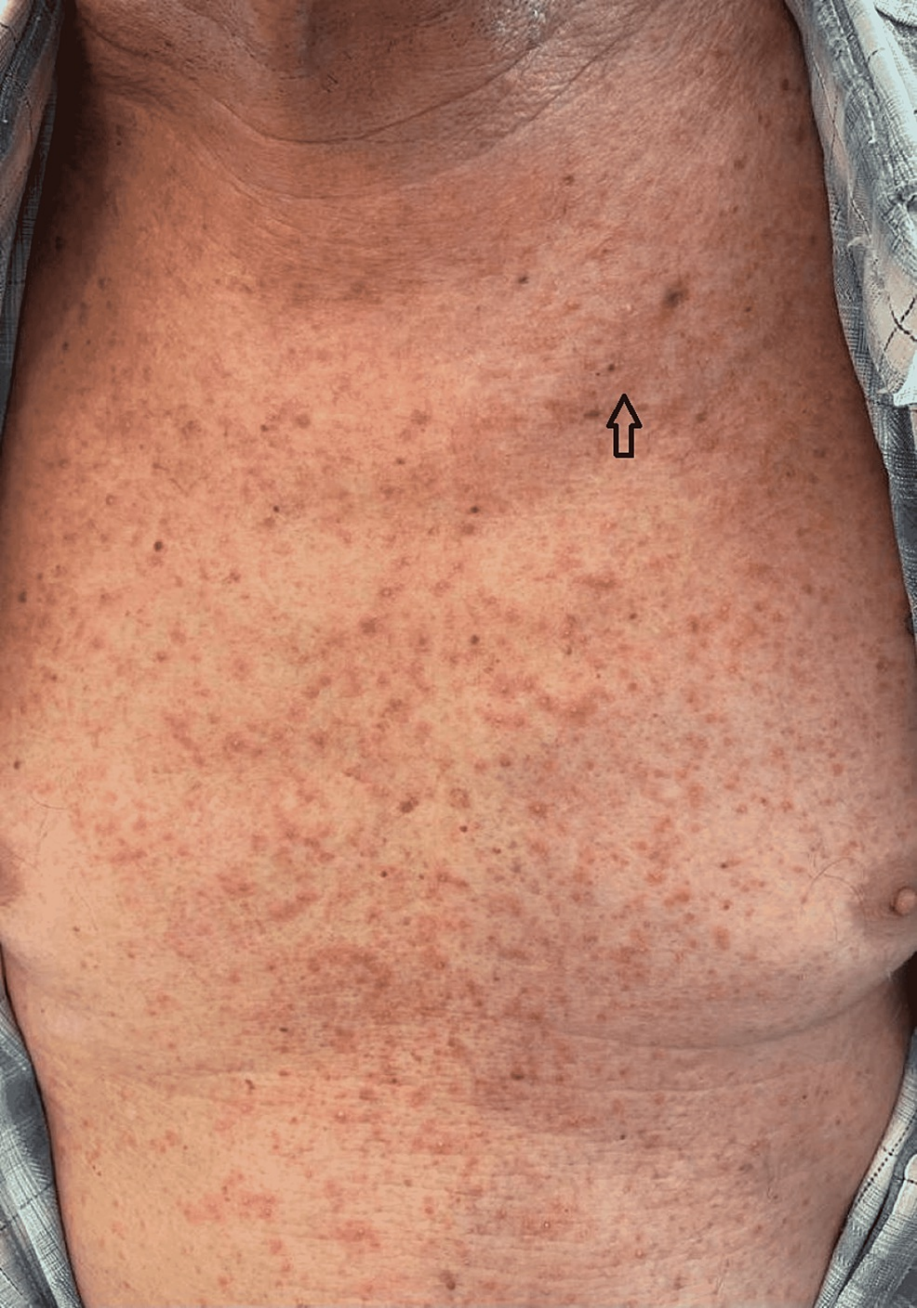
Papulopustular rash on anterior thorax

**Figure 3:**
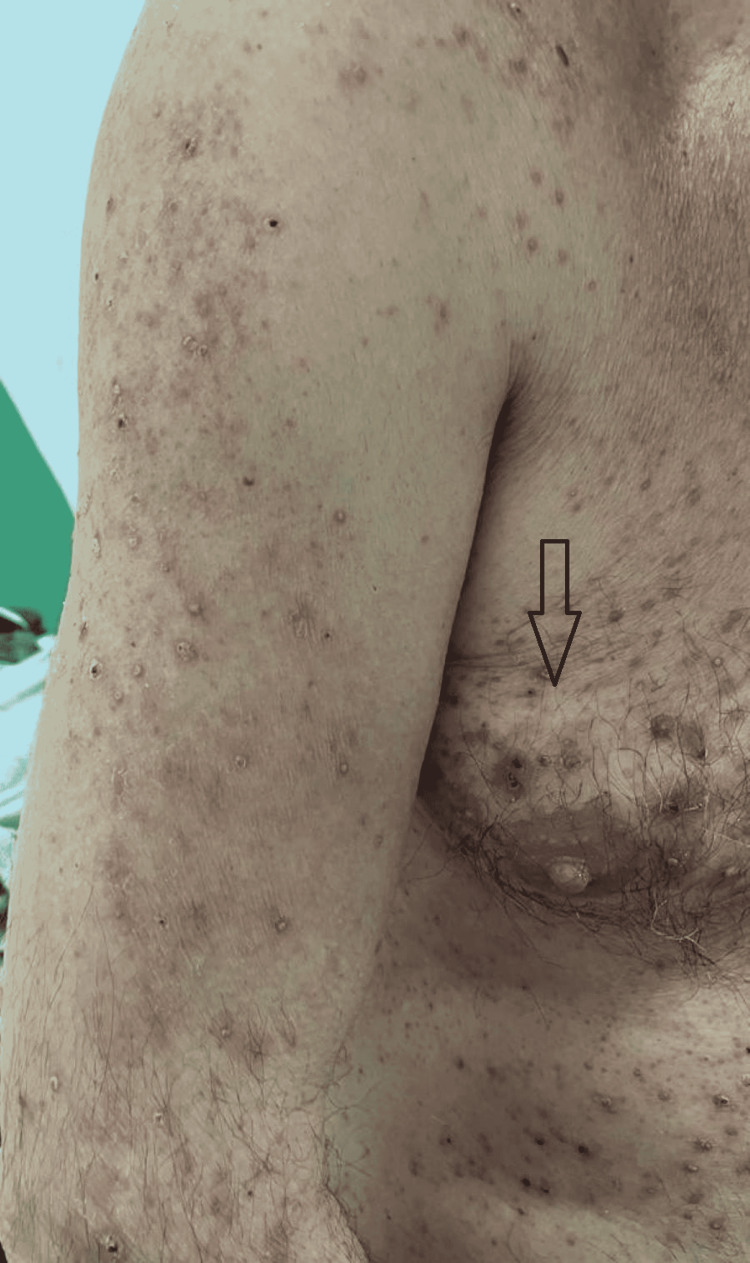
Papulopustular rash, xerosis, hematic crusts, hyperpigmentation

**Figure 4:**
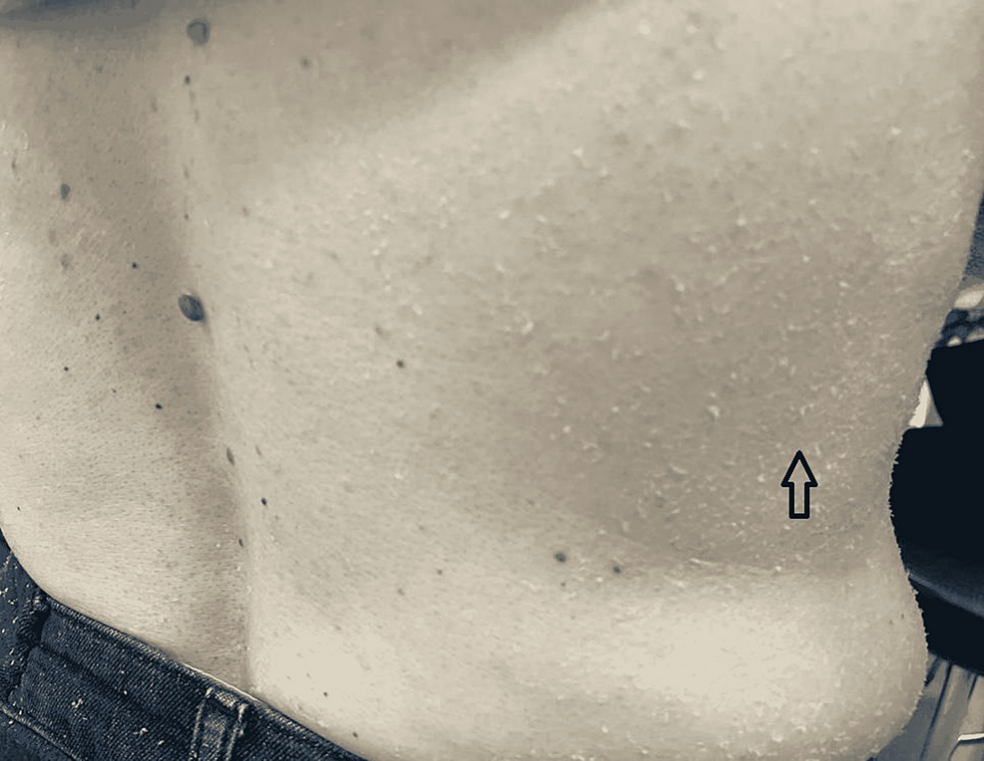
Xerosis and scales

**Figure 5:**
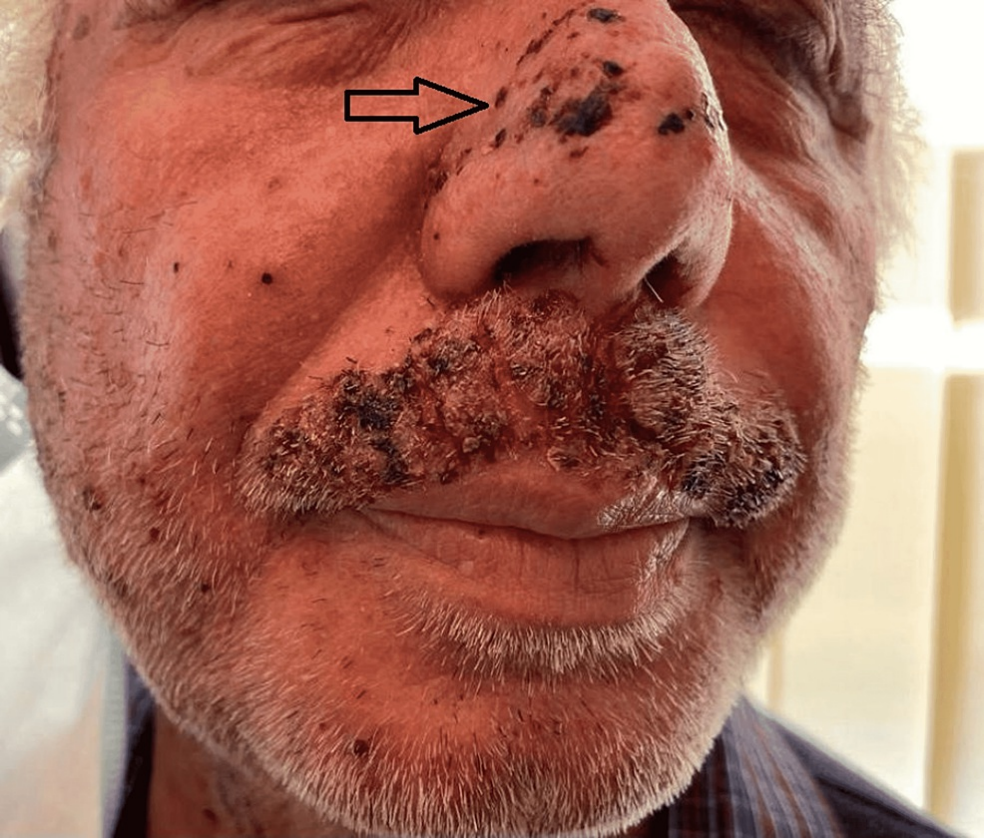
Hematic and meliceric crusts, superinfections

**Figure 6:**
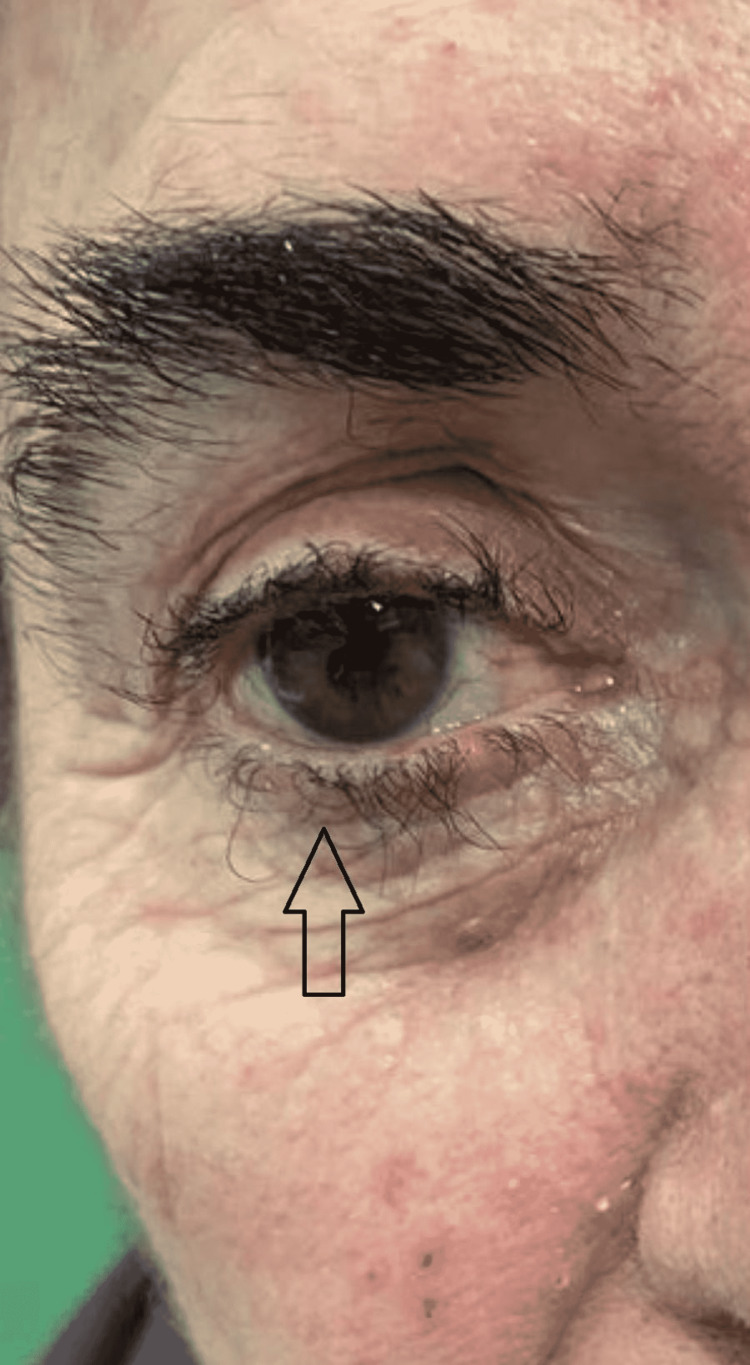
Trichomegaly

**Figure 7:**
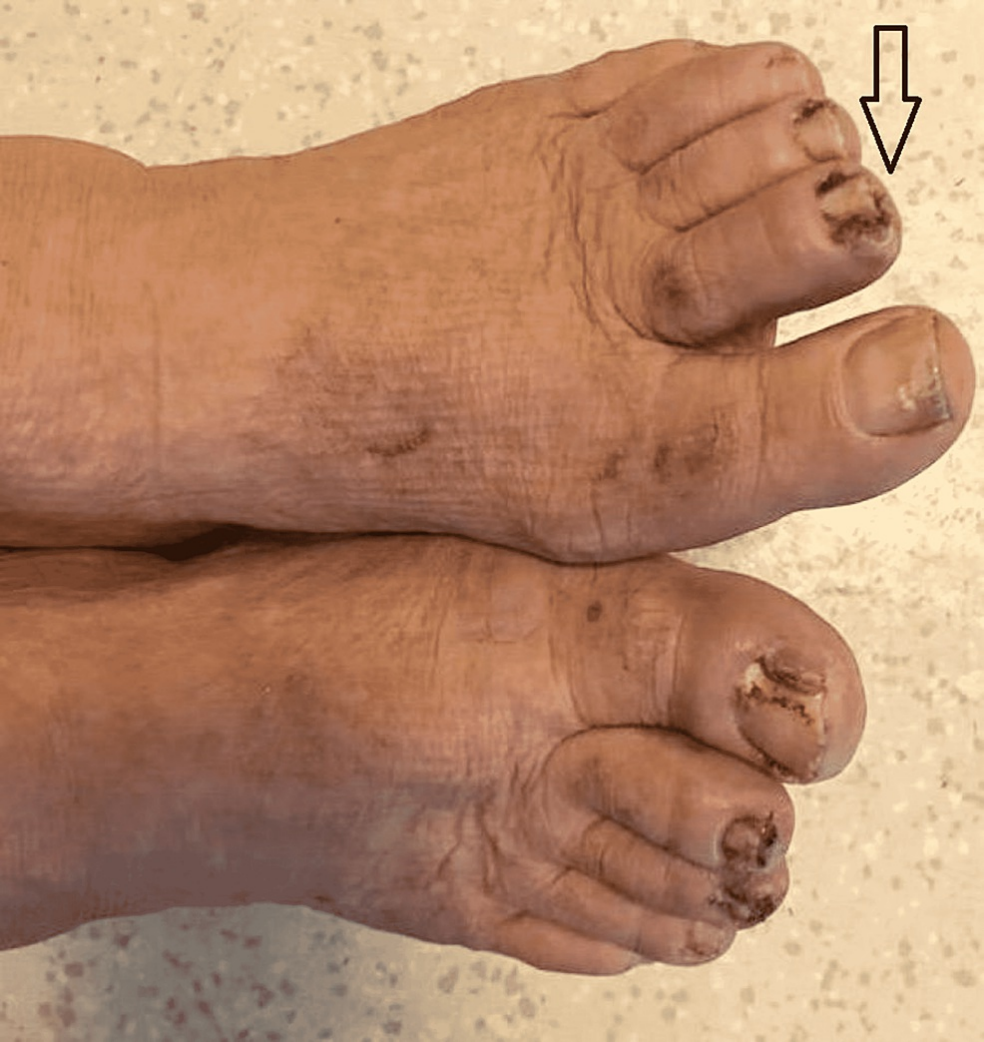
Nail changes: paronychia in combination with periungual pyogenic granuloma

**Figure 8:**
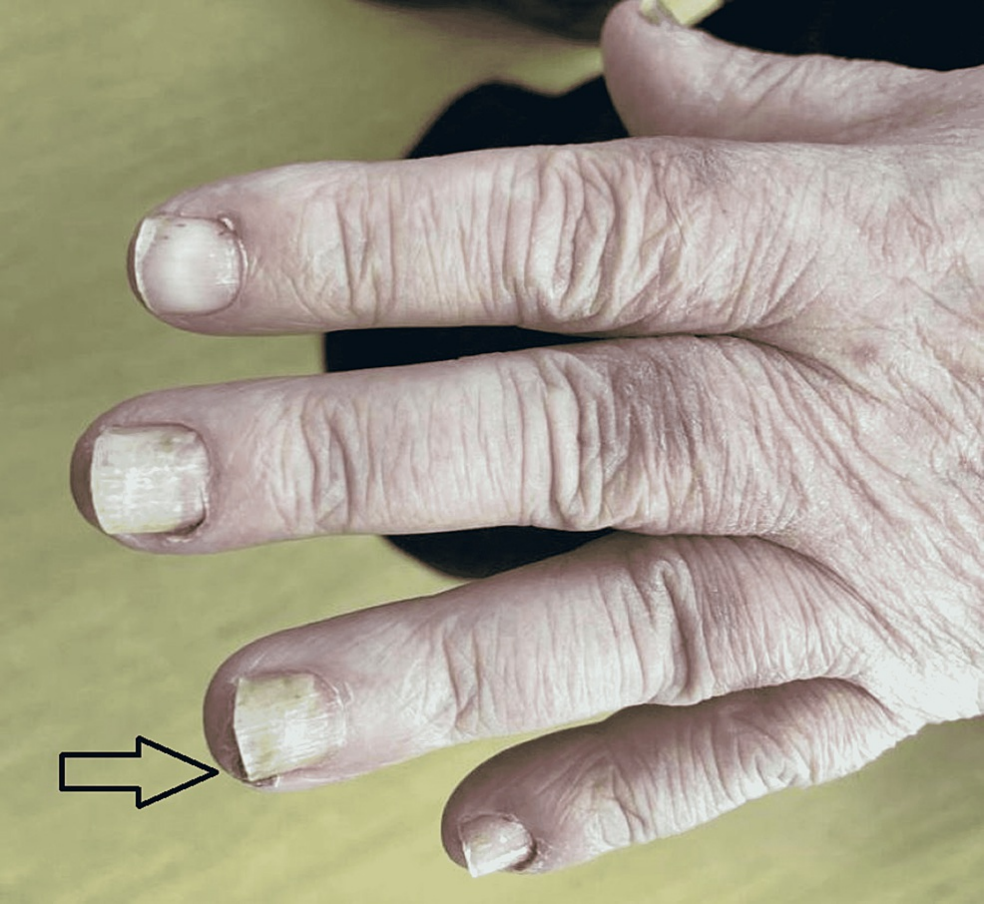
Nail changes: granuloma, onychomycosis, cracks, panaritium

In this study group of 10 patients, we identified dermatological side effects such as papulopustular rashes (Figures 1-3) (more frequently on the face, anterior and posterior thorax, and upper limbs, and less often, on the abdomen and lower limbs), facial erythema, telangiectasia, hyperpigmentation, followed, with a lower incidence, by xerosis (Figure 4), pruritus, hematic and meliceric crusts (Figure 5), photosensitivity, flushing, and eczema. We also identified hair changes that consisted of localized hair loss (alopecia), hypertrichosis, and morpho-functional changes in structure and color, becoming wavy and curly. The eyelashes became lengthened and curly (trichomegaly; Figure 6) causing blepharitis and conjunctivitis. Furthermore, a wide range of nail changes (Figures 7-8) has been identified, including cracked lateral nail folds, paronychia, panaritium, onycholysis, periungual granulomas, fissures, and onychomycosis.

Feces culture

The fecal samples (minimum 1 g) were collected in a container without a transport medium and transported to the laboratory at 2-8° C in order to be cultured. The samples were secured and transported to Germany (Ganzimmun Diagnostics AG, Mainz) for gut flora analysis by fecal culture and identification of the dysbiosis degree.

Only patients who had not received antibiotic or probiotic treatment in the last 14 days prior to collection and patients who had no dietary changes were included in the study.

Following the laboratory analysis, we performed cultures for putrefactive microbiome flora (Escherichia coli, Escherichia coli-types, Proteus spp., Klebsiella spp., Enterobacter spp., Serratia spp., Morganella morganii, Citrobacter spp., Pseudomonas spp., Enterococcus spp., Staphylococcus aureus, Salmonella, and Shigella), protective flora (Bacteroides spp., Bifidobacterium spp., Lactobacillus spp., Clostridium spp., and Clostridium difficile), and fungi (Candida albicans, Candida non-albicans, Geotrichum spp., and molds).

## Results

In order to determine intestinal dysbiosis degree in the two groups under study, the analysis of the intestinal microbiome consisted of the analysis of the putrefactive flora, the protective flora, and the identification of the presence or absence of fungi in the feces.

**Figure 9:**
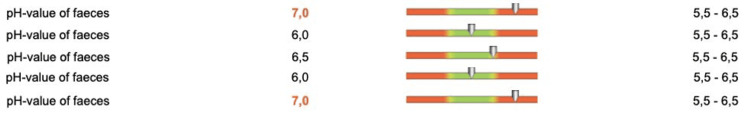
pH of the feces in patients treated with doxycycline for two weeks

**Figure 10:**
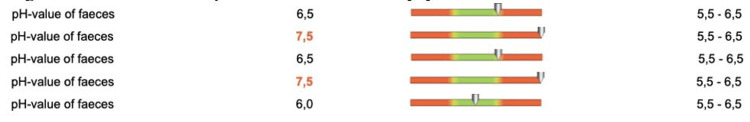
pH of the feces in patients treated with doxycycline for eight weeks

In terms of the pH values of feces, there were no significant changes between patients receiving short (less than two weeks) (Figure 9) and patients receiving long-term doxycycline treatment (eight weeks) (Figure 10).

Group 1: patients undergoing EGFRI treatment and doxycycline for less than two weeks

**Figure 11:**
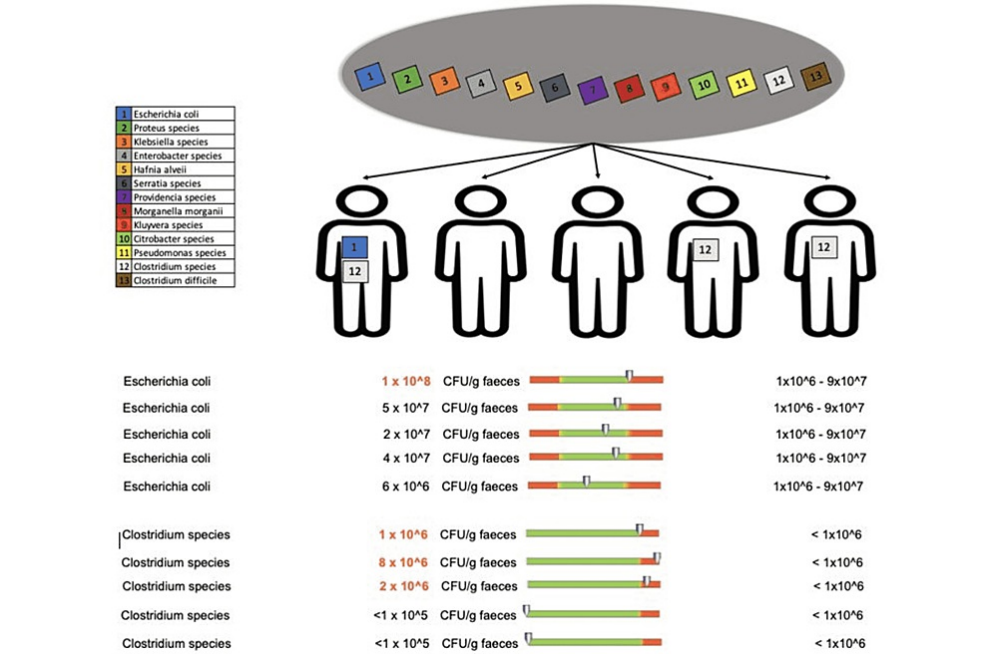
Changes in the putrefactive flora in patients who received doxycycline for less than two weeks

Patients who received doxycycline treatment for less than two weeks showed changes in the putrefactive flora for only two bacterial species: Escherichia coli in one out of five patients and Clostridium species in three out of 12 patients (Figure 11).

**Figure 12:**
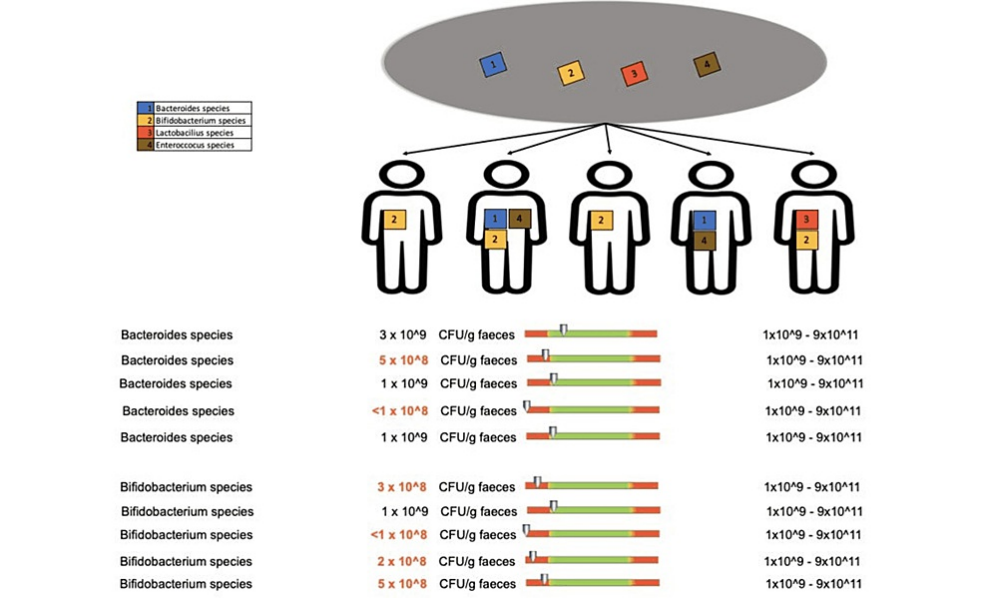
Changes in the protective flora (Bacteroides species and Bifidobacterium species) in patients who received doxycycline for less than two weeks

**Figure 13:**
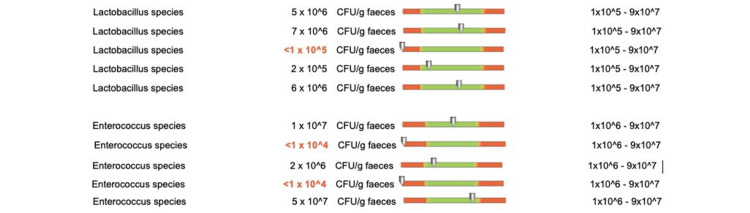
Changes in the protective flora (Lactobacilli and Enterococcus species) in patients who received doxycycline for less than two weeks

Regarding the protective flora of patients who underwent doxycycline treatment for less than two weeks, we recorded changes in Bacteroides species in two out of five patients, Bifidobacterium species in four out of five patients (Figure 12), Lactobacilli in one out of five patients, and Enterococcus species in two out of five patients (Figure 13).

**Figure 14:**
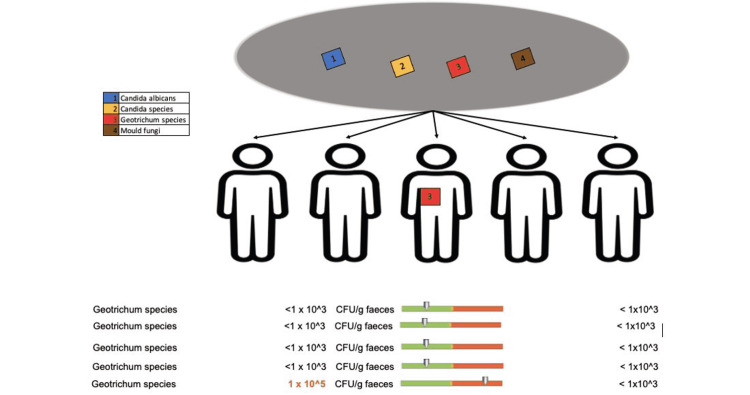
Changes in the fungal composition in patients who received doxycycline for less than 2 weeks

Regarding the fungal composition, only one patient who received doxycycline treatment for less than two weeks showed changes in Geotrichum species (Figure 14).

Group 2: patients undergoing EGFRI treatment and doxycycline for more than eight weeks

**Figure 15:**
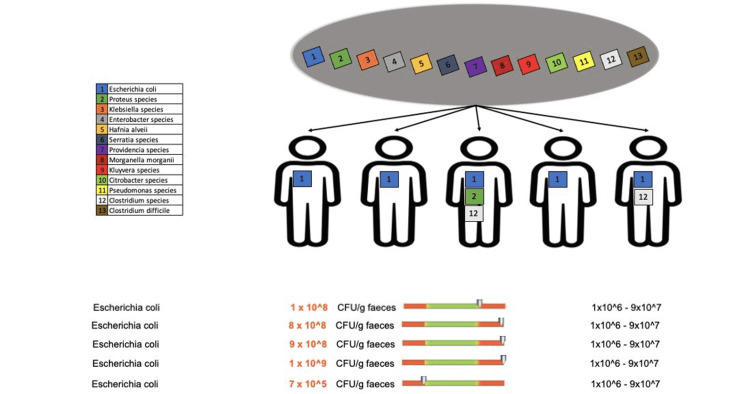
Changes in the putrefactive flora (E. coli) in patients who received doxycycline for more than eight weeks

**Figure 16:**
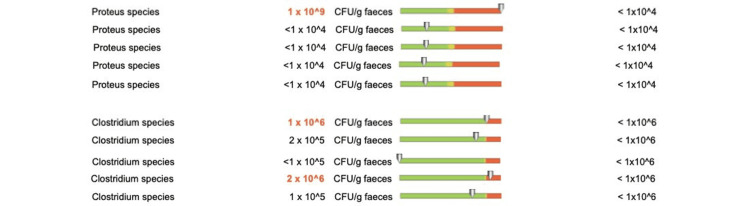
Changes in the putrefactive flora (Proteus species and Clostridium species) in patients who received doxycycline for more than eight weeks

We observed changes in the putrefactive flora of patients undergoing EGFRI treatment and doxycycline for more than eight weeks. All patients showed overexpression of E. coli (Figure 15), one out of five patients showed increased values of Proteus species, and two out of five patients showed changes in Clostridium species values (Figure 16).

**Figure 17:**
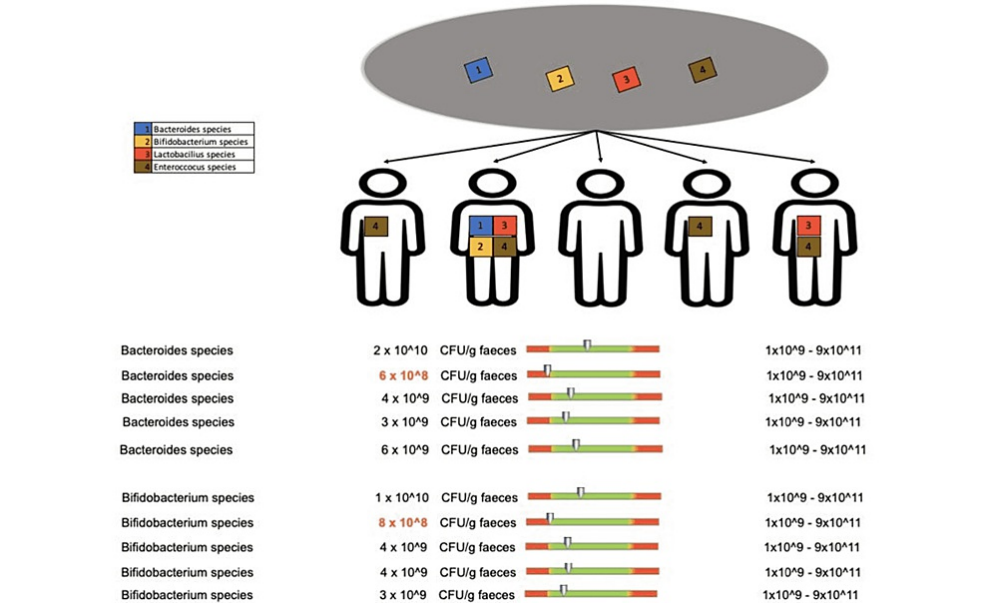
Protective flora changes (Bacteroides species and Bifidobacterium species) in patients who received doxycycline for more than 8 weeks

**Figure 18:**
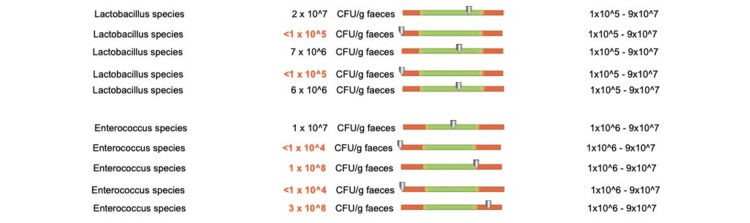
Protective flora changes (Lactobacilli and Enterococcus species) in patients who received doxycycline for more than eight weeks

Regarding the composition of the protective flora, patients who received doxycycline treatment for more than eight weeks experienced value changes of Bacteroides species in one out of five patients, Bifidobacterium species in one out of five patients (Figure 17), Lactobacilli in two out of five patients and Enterococcus species in four out of five patients (Figure 18).

**Figure 19:**
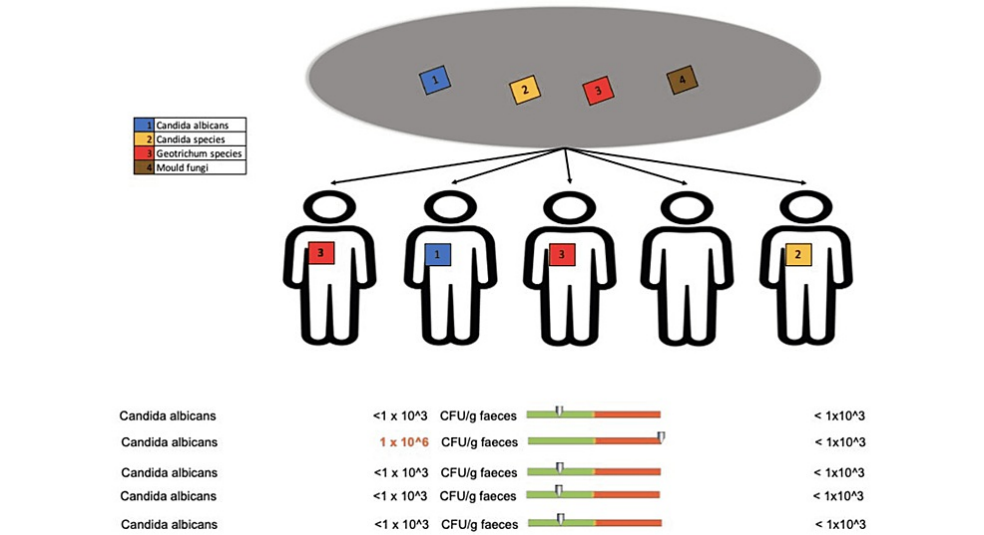
Fungal species changes (Candida albicans) in patients who received doxycycline for more than eight weeks

**Figure 20:**
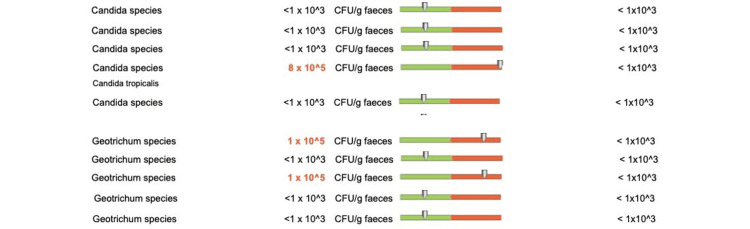
Fungal species changes (other Candida species and Geotrichum) in patients who received doxycycline for more than eight weeks

In terms of the fungal composition, we recorded overexpression of Candida albicans species in one out of five patients (Figure 19), other Candida species in one out of five patients, and Geotrichum in two out of five patients (Figure 20).

## Discussion

The current study aimed to identify the totality of compositional and diversity changes in the gut microbiome, as well as the assessment of dysbiosis, following antibiotic (doxycycline) treatment in patients with colorectal cancer and skin toxicity undergoing EGFRI treatment.

At the moment, there is no specific guideline for the management of dermatological reactions occurring in patients undergoing EGFRI treatment. The use of antibiotic treatment is either prophylactic or reactive.

Patients with CRC often present imbalances in the intestinal flora. In addition to genetic factors, environmental factors, such as a high-fat diet, fiber deficiency, red meat consumption, lack of physical activity, infectious agents, surgery, or antibiotics, are involved in the etiology of CRC. The presence of these environmental factors ultimately leads to changes in the gut microbiome [12].

The use of antibiotic treatment causes many changes in the intestinal flora. Most commonly, these changes consist of a decrease in the beneficial, protective bacteria accompanied by overexpression of the pathogenic or putrefactive flora [13].

In this study, we highlighted the variations in the intestinal bacterial and fungal flora found in cancer patients undergoing EGFRI treatment and doxycycline for short or long periods of time. All patients treated with doxycycline for eight weeks showed increases in the Escherichia coli (E. coli) values.

The occurrence of the inflammatory processes in the gastrointestinal tract as a result of infections, antibiotic treatment, or dietary changes leads to intestinal dysbiosis characterized mainly by an increase in enterobacterial species [14].

The Enterobacteriaceae group consists of species such as E. coli, Enterobacter, Klebsiella, Proteus, Citrobacter, Pseudomonas, Serratia, Morganella, Hafnia, and Yersinia. High values associated with these species in the environment may justify their presence in the feces of healthy people following food consumption. However, elevated values of these species (more than 10Ù5 KBE/g feces) indicate disturbances in the intestinal flora.

Increased levels of Escherichia coli can result in the release of considerable amounts of metabolites. When an increase in the carbohydrate release occurs, flatulence and meteorism are observed. In case of increased protein substrate release, E. coli causes changes in the form of amines (histamine, putrescine, and tyramine) and ammonia, which have toxic effects on the liver.

Overexpression of Escherichia coli was also recorded in the colorectal cancer patients who were treated with doxycycline in this study. All patients undergoing long-term (eight weeks) doxycycline treatment showed dysbiosis phenomena of the putrefactive flora. This event is characterized by the overexpression of E. coli and was correlated to the long-term use of doxycycline since only one patient from the short-term (two weeks) group showed a slight increase in E. coli levels.

Patients with a longer duration of the antibiotic treatment (group 2) also experienced overexpression of fungal populations. More specifically, we recorded increased levels of Candida albicans, other Candida species, and Geotrichum species in four out of five patients. Similar to the E. coli analyses, this event was correlated to the prolonged use of the antibiotic, with only one patient from the short-term doxycycline treatment group showing overexpression of the Geotrichum species.

The protective flora is made up of a high proportion of bacterial species such as Bifidobacterium, Bacteroides, Lactobacillus, and Enterococcus. Most of the bacterial species belonging to the protective flora are included in the composition of probiotics, underlining their importance in maintaining good health [15].

The presence of normal values of Bifidobacterium species has been associated with the occurrence of T-cell-mediated anti-tumor response. Among the subspecies with these properties, Bifidobacterium adolescentis, Bifidobacterium breve, and Bifidobacterium longum, which are known to have a strong effect on immunity, have been identified [16].

Numerous studies highlight the ability of Bifidobacterium species to modulate and stimulate the host immune response, including both the immune defense and the acquired immunity [17].

Alongside Bifidobacterium, certain Bacteroides species, known as "anti-cancer probiotics," have now been identified, and they form the bulk of the colonic flora. Bacteroides species have a beneficial, protective effect by metabolizing oligosaccharides and polysaccharides, with the purpose of producing vitamins and nutrients needed by the host [13].

In adults, the number of Bacteroides species can also be influenced by environmental factors, antibiotic treatment, and the host’s diet [18].

Another extremely important component of the protective flora is Lactobacillus, so the low levels of Lactobacillus species increase the risk of putrefactive bacteria and external agents overgrowth. Lactobacilli also play an important role in acidifying the intestinal flora [19].

Another marker of intestinal dysbiosis is the decreased levels of Enterococcus, with normal values of Enterococcus species representing a marker for assessing the stability of the protective flora. Its deficiency increases the external risk of the development of infections and colonization with pathogens [20-21].

Both groups of patients included in this study showed acute dysbiosis of the species defining the protective flora.

In the group of patients with long-term antibiotics treatment (8 weeks), a greater deficit of Enterococcus species was observed compared to patients who underwent antibiotic treatment for only two weeks.

The main limitation of this study is the relatively small number of patients. Another limiting factor is represented by the lack of microbiome analysis before the start of the antibiotic treatment, which would have enabled us to dynamically highlight the totality of changes in the gastrointestinal microbiome.

This analysis is a personal contribution to the study of the gut microbiota in colorectal cancer patients undergoing EGFRI treatment and experiencing skin toxicity. We identified the bacterial and fungal species that cause intestinal dysbiosis and highlighted dysbiosis following treatment with doxycycline, which is widely used in these patients. Understanding these data will facilitate the choice of the prophylactic or reactive treatment with doxycycline and therefore the duration of the antibiotic treatment in patients with skin toxicity undergoing EGFRI treatment.

## Conclusions

The use of the antibiotic treatment has profound and sometimes persistent effects on the gut microbiota by depleting beneficial species and raising the levels of pathogenic species, especially in colorectal cancer patients with already altered gut flora.

The overexpression of Escherichia coli and candida species in patients who received doxycycline for a longer period of time compared to patients who received it for two weeks could represent the basis for the development of a standardized guideline for the use of pre-reactive or reactive antibiotic treatment. We also highlight the importance of analyzing the intestinal microbiome of these patients. The identification of overexpressed species, as well as of the deficiency of specific protective species could guide the administration of probiotics to cover and repair the affected gut microbiota.
